# What Is the Best Strategy for Reducing Deaths from Heart Disease?

**DOI:** 10.1371/journal.pmed.0020098

**Published:** 2005-04-26

**Authors:** Michael E Makover, Shah Ebrahim

## Abstract

Background to the debate: Coronary artery disease is a major cause of death worldwide. Two very different approaches have been proposed as a way of reducing these deaths. The “high risk” approach uses tools such as risk factor scoring and carotid ultrasound to try and identify those at highest risk, and then treats them aggressively. The “population” approach aims to shift the distribution of risk factors across a population in a beneficial direction with the goal of reducing heart disease in the whole population.

## Abstract 2

Should we identify and treat only those at highest risk, or should we try to shift the distribution of risk factors across the whole population?

## Michael Makover's Viewpoint: We Should Use High-Sensitivity Carotid Ultrasound to Detect Very Early Atherosclerosis and Treat Aggressively

**Figure 1:**
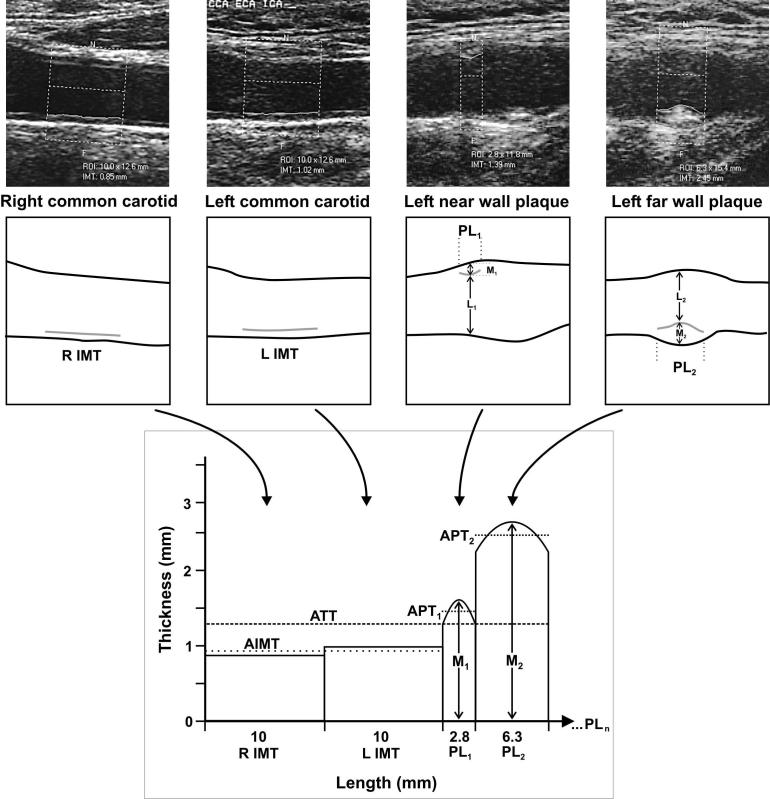
Ultrasonography of the Carotid Artery to Measure the IMT This figure provides a framework for understanding the multiple, quantitative calculations undertaken for each patient's carotid ultrasound scan. Two images of carotid artery without plaques (left) and two with plaques (right) are shown at the top. ROI indicates the region of interest in which thickness measurements are made. These were obtained from a single patient. Schematics for each image are provided below the video images. The grey lines indicate the measured IMT within the defined region of interest. Regarding the plaques (PL_1_ and PL_2_), M indicates maximum plaque thickness, and L indicates minimum lumen diameter at the site of maximum plaque thickness. Percent diameter stenosis is calculated as [M/(M + L)] × 100%. The graph (bottom) indicates length in millimeters on the x-axis and thickness in millimeters on the y-axis. The areas from the far wall of the left (L) and right (R) common carotid arteries are measured in all patients. AIMT designates the average IMT derived from the left and right measurements. PL_1_ and PL_2_ are the lesion lengths of the respective plaques, and M_1_ and M_2_ are the respective maximal thicknesses. APT_1_ and APT_2_ designate the respective average lesion thicknesses for each plaque. The total area is given by the sum of all the areas shown on the graph in units of square millimeters. The average total thickness (ATT) is given by the total area divided by the total length shown on the x-axis. PL_n_ on the x-axis is intended to indicate that the calculations are undertaken using these concepts, irrespective of the number of plaques identified in a given study. (Figure from [28] with permission of all four authors.)

Atherosclerotic vascular disease is the greatest cause of death and disability in developed countries. The best way to prevent these outcomes is to detect disease at the earliest possible stage and to attack it with pinpoint-targeted aggressive treatment. B-mode ultrasonography of the carotid artery to measure the intima-media thickness (IMT) (Figure 1) is the most effective way to do that.

It is time to end the mindset that thinks of atherosclerosis as an inevitable function of aging. It is not: it is a disease, whether it causes an acute event or gradual decline. As with any disease, the earlier and more intensively we attack it—with medications and lifestyle changes—the more successful we will be in containing and reversing it. Ultrasonography of the carotid to detect plaque and increased intimal wall thickness is the best, safest, and easiest early detector we have.

Patients at risk for atherosclerotic cardiovascular disease are identifiable only up to a point using traditional methods, including the Framingham risk score [1], the European SCORE (Systemic Coronary Risk Evaluation) [2], and the C-reactive protein level [3]. These are particularly effective for people in the highest risk groups, but they have serious flaws. They do not take family history of premature coronary artery disease into account; they do not benefit from new assays that can differentiate lipoproteins by particle size and number, both of which are important factors in atherogenicity; and they do not include the quality of the patient's diet, abdominal fat content, racial and ethnic genetic differences, confounding medical conditions, certain gender differences, and other factors. Thus, many patients deemed “low risk” for acute events within a period of time might still develop disease.

All of the current risk models were developed within the obsolete gestalt of life spans of perhaps 60 to 70 years, whereas people are now increasingly living productively into their 80s and 90s. Risk tables are based only on cardiac events over a relatively short time period of ten years. Progress in medical science is now on the steepest slope of exponential growth. It is reasonable to expect that those who maintain healthy circulation will experience good quality life beyond the 100-year mark in the surprisingly near future, and perhaps to 120 years not long after. Furthermore, risk escalates with age. Thus, an initial low risk profile becomes far more significant when considered in the context of long lives. Equally important, gradual impairment of blood flow that nourishes tissues can lead to peripheral vascular disease, increased susceptibility to infection, cognitive decline, frailty, and other changes that impair life quality and longevity.

While risk factors help individualize treatment, they do not address the most critical issue: identifying atherosclerosis at the very earliest stage to try to stop and reverse the process *before* any damage has occurred. Risk factors identify those who *might* develop disease. The “smoking gun” is to identify disease when it actually first develops. Atherosclerosis begins as a lipid and inflammatory cell deposition in the intimal subendothelial space, which expands outward, with only a slight inward intrusion on the lumen. This does not affect blood flow unless the lumen-side plaque cap ruptures, and a clot forms and expands into the blood stream. Most clots are limited by thrombolytic mechanisms, but significant blockage usually results as the clot is scarred over. Some clots expand all the way across to the other side of the artery, completely occluding it, causing a heart attack or stroke [4].

Thus, if we could discover plaque at the very earliest stage, we could aggressively attack it with our full armamentarium of lifestyle modification, addressing all the factors noted above, and medications—statins, ACE inhibitors, aspirin, niacin, ezetimibe, and others—to stabilize and then reverse the process before a rupture occurs. The REVERSAL trial, among many others, has shown that atherosclerosis is reversible with aggressive treatment [5]. Carotid ultrasound is an easy, safe, noninvasive method for detecting early, focal plaques and early thickening of the inner lining of the artery [6]. There is ample evidence that this is an effective and reproducible detector, and a predictor of progression and symptomatic disease [7]. In contrast, angiography is invasive and is insensitive to early changes in the artery wall thickness. Magnetic resonance imaging is expensive, cumbersome, and still experimental. Calcium scoring by computed tomography is less sensitive and less reproducible and suggests plaque presence only indirectly [8]. Angiography and calcium scoring entail considerable X ray exposure.

Using high-sensitivity carotid ultrasound as a primary screening tool would add modest cost, but that would be more than offset by sharply reducing the enormous worldwide toll of atherosclerosis. Screening tests should be easy, affordable, widely available, and predictive. Carotid ultrasound meets all these criteria and should be used to screen everyone early on who is at any increased risk by measure of the expanded risk factors noted above.

## Shah Ebrahim's Viewpoint: Reducing Risk Factors across the Population Is Better Than Identifying Only Those at Highest Risk

**Figure 2:**
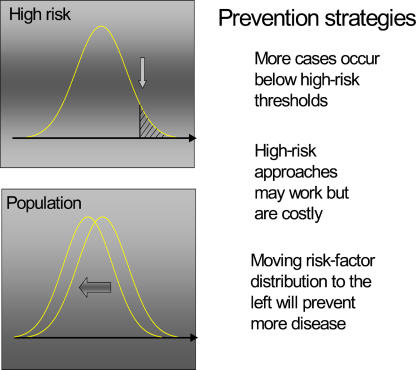
The High Risk Versus the Population Approach to Prevention (Figure: John Emberson)

The “high risk” approach to reducing heart disease involves identifying those at highest risk by means of risk factor scoring, dominated by the Framingham equations [9], followed by aggressive risk factor control. This approach must be contrasted with the “population” approach to prevention (Figure 2), which aims to shift the whole distribution of risk factors in a beneficial direction and thereby reduce the incidence of cardiovascular disease in the whole population. In the population approach, all have to take part in making the population healthy but only relatively few will benefit—the “prevention paradox” [10].

Population change does require political commitment—as Virchow commented, “Mass diseases require mass solutions” [11]. There are several politically difficult population actions that many public health agencies would wish to implement to reduce heart disease. These include extension of restrictions on the use and sale of tobacco products, reduction of “hidden” saturated fats in processed foods, and an increase in the opportunities for regular physical exercise. Some of these policies cut across the vested interests of international corporations that have powerful means of controlling elected governments, leading to a laissez-faire approach of emphasising individual “choice” as the means of improving public health [12].

Although stopping smoking and lowering blood pressure and blood cholesterol are beneficial, the crucial question is whether using risk factor screening tools to identify people at high risk for disease and then treating them is more effective than focusing on individual risk factors. In common with many screening enterprises, there is only limited evidence to help determine whether screening for those at highest risk of heart disease is worthwhile. This reflects both the difficulty of mounting trials of screening (which are costly, large scale, and of long duration) and the “culture” of evaluation of screening, which has been dominated by Wilson and Jungner's out-dated criteria that do not emphasise the fundamental importance of randomised trials in evaluation [13]. Screening, like any other medical technology, requires robust evaluation [14].

The evidence available from randomised controlled trials of the high risk approach is not encouraging. The impact on clinical events in community populations is very limited—the best estimate is of no real effect at all, although an effect as small as a 10% risk reduction may have been missed [15]. Since medications for reducing coronary disease morbidity and mortality are clearly effective, it is tempting to look for better screening tests for early disease, such as carotid ultrasound with plaque characterisation, fast computed tomography scanning of the coronary arteries, and genetic “SNPing” (looking for single nucleotide polymorphisms associated with coronary disease) [16].

But none of the new screening technologies currently recommended [17] have been adequately assessed in randomised controlled trials. Ultrasound IMT measures are capable of identifying people at increased risk. However, in evaluations of the receiver–operator curves comparing the accuracy of ultrasound measures with conventional risk factor scoring, no extra predictive power is obtained from ultrasound [18,19]. This is not surprising as increased IMT and plaque are simply the downstream consequences of conventional risk factors operating over many years.

Even if screening tools were highly accurate, high risk approaches can never match the health gains achieved by small downward movements in the distribution of risk factors. This is because the high risk group is (by definition) small relative to the rest of the population and consequently even though these people are at higher relative risk, the absolute numbers of coronary events will always be greater in the much larger “low risk” population. Of course, one way around this problem is to define the whole population as “high risk” and give everyone several medications (a “polypill”) to lower their risk [20].

Estimates of how much can be achieved by treating all those at a 15% or greater risk of suffering a cardiovascular event over 10 years (about half the older adult population) with a combination of effective drugs, show that, at best, only *half* of the events would be prevented. By contrast, small downward movements in blood pressure and blood cholesterol of 10%–15% of the mean will reduce coronary heart disease events by the same amount [21]. Improving the accuracy of risk prediction with new screening technologies will not alter this relationship between high risk and population approaches.

If cost is factored into any policy-making process, it is obvious that investing in widespread use of new screening technologies will, inevitably, be less cost-effective than implementing population policies. In privatised, individualised, health-care systems of the United States it is much more likely that new screening developments will arise without evaluation and will prove popular with naïve, but worried, well people. In these circumstances, the onus is on the scientific community to demand better standards of evidence, as many governments will not.

## Makover's Response to Ebrahim's Viewpoint

While the “population” approach is obviously very important, there is no reason not to use the “high risk” approach as well. “High risk” is the wrong term—*all* arteries matter over a 120-year life span. We must attack atherosclerosis at the very onset, not wait until it is “high risk.” All previous approaches were designed to detect those at allegedly higher risk by various criteria. This misses the point. Once plaque exists, risk exists, especially when viewed over a full lifetime, not the current ten-year standard. And although the aggressive approach I outlined in my viewpoint would obviously have a cost, the savings (a reduction in the financial toll of cardiovascular disease) would be greater than the cost.

Ultrasonography of the carotid artery intimal wall is effective and highly predictive according to the great preponderance of studies [8]. Ebrahim cites two studies to support his argument that measuring carotid IMT offers no additional predictive power over conventional screening tools [18,19], but the first of these has serious flaws (such as a significant dropout rate that the authors assumed was random, a short follow-up period, and the inclusion only of people of an advanced age) [18], and the other concluded that carotid IMT measurement “substantially improved prediction of future coronary heart disease” [19].

Millions of people are at risk now and cannot wait for endless studies to prove over and over what we already know. It is not “naïve” for well people to want to remain well and to maintain their arteries to enable healthy older lives. Nor should we disparage as “worried” the very human desire to remain that way. Population-wide planning is important, but doctors take care of each patient as an individual. Carotid ultrasonography is by far the best way to design treatment for every patient exactly as needed. I believe that there is already sufficient evidence and sensible rationale to meld already well-proven technologies and approaches into a comprehensive, aggressive attack on atherosclerosis.

## Ebrahim's Response to Makover's Viewpoint

Declines in the risk of coronary heart disease have been reported in many countries that had experienced cardiovascular disease “epidemics” during the middle of the 20th century [22]. The onset of these beneficial trends pre-dated the introduction of both risk factor screening and widespread use of effective treatment of hypertension and hypercholesterolaemia. These downward trends in cardiovascular mortality have contributed to increases in life expectancy.

In autopsies done during the Korean and Vietnam wars, atherosclerosis was found in 77% and 45% of young men, respectively [23,24]. More recently, the origins of cardiovascular disease have been pushed back to fetal development and early childhood [25,26]. Raised levels of risk factors are set in early life, and if earlier intervention means better outcomes, it is at this stage (i.e., in early life) that treatment and prevention must be targeted.

Plaques are remarkably common in middle-aged people screened using ultrasound [27], but most are stable and unlikely to rupture. Presence of plaque in otherwise healthy people does not identify those who are destined to suffer an event or benefit from treatment. In the same way as risk factors provide a probabilistic evaluation of the chances of suffering a clinical event, so too does presence of plaque, with no greater accuracy than risk factor scoring methods [19].

Using carotid ultrasound screening to define a subgroup of those with plaque but normal or low levels of cardiovascular risk factors may be useful (see Table 1). However, studies have yet to be done to confirm whether plaque, in the absence of raised risk factors, is harmful. Importantly, trials are needed to determine whether treating such people aggressively—lowering their already low risk factors—results in better outcomes. It is possible that imaging of arteries may provide extra psychological incentives for patient adherence to lifestyle and drug regimens, but this too remains to be tested in well-designed randomised trials.

**Table 1:**
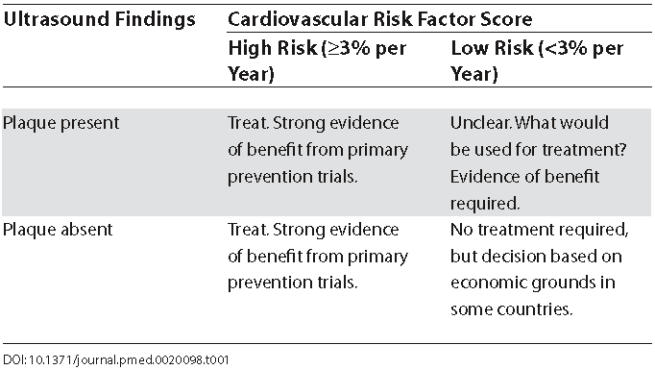
Treatment Decisions in Relationship to Carotid Ultrasound and Cardiovascular Risk Screening: Will Ultrasound Be of Value in Deciding Whether Some Low Risk Patients Should Be Treated?

## Abbreviation

IMT: intima-media thickness
